# Management of Aberrant Frenal Attachments in Adults by Scalpel Method and 980 nm Diode Laser

**DOI:** 10.1155/2023/7258637

**Published:** 2023-01-04

**Authors:** Jaahnavi Lanka, Pratibha Gopalkrishna, Santhosh Kumar

**Affiliations:** ^1^Department of Periodontology, Army College of Dental Sciences, Secunderabad, Telangana 500087, India; ^2^Department of Periodontology, Manipal College of Dental Sciences, Manipal, Manipal Academy of Higher Education, Manipal, 576104, Udupi, Karnataka, India

## Abstract

Treatment modalities like electrosurgery and lasers have emerged as effective painless alternatives to scalpel methods for a frenectomy. The present case series involves ten patients, diagnosed with abnormal frenal attachments. Frenectomy was performed by 980 nm diode laser and scalpel methods. Scalpel frenectomy was performed as described by Archer and Kruger. Laser-assisted frenectomy was performed by a hemostat-guided incision using a 980 nm diode laser tip in a paintbrush motion. It was observed that Visual Analogue Scale (VAS) pain scores in patients who underwent scalpel frenectomy were comparatively higher than the laser-treated patients. In contrast, wound healing scores were higher in the scalpel group, suggesting early wound healing in the scalpel-treated patients.

## 1. Introduction

Aberrant frenal attachments are minor congenital malformations [1, 2] that can lead to unpleasant esthetic problems, such as midline diastema [1, 2]. The management of these abnormal frenal attachments poses clinicians a challenge, as patients delay frenum corrections due to fear of the procedure. Furthermore, the delay in correcting these abnormal frenal attachments puts the patients at risk of compromised oral hygiene, gingival recession, bone loss, periodontitis, and eventually tooth loss [3, 4].

Postoperative bleeding, pain, and speaking discomfort are a few unpleasant events associated with conventional scalpel methods [5–10]. To overcome these difficulties and increase patient compliance and acceptance, newer methods like electrosurgery and lasers have come into use [5–10]. Diode laser was chosen for its suitability for soft tissues, anti-inflammatory, and bactericidal properties [11]. However, randomized clinical trials comparing scalpel frenectomy to 980 nm diode laser-assisted frenectomy using appropriate pain scales and wound healing indices are limited. Hence, the present case series aims to closely observe patient-reported pain perception and wound healing patterns in scalpel and 980 nm diode laser-assisted frenectomy.

## 2. Presentation of Case Series

P. Mirko et al. [3] identified four types of frenal attachments as—mucosal (frenum attached at the mucogingival junction), gingival (frenum attached within the attached gingiva), papillary (frenum attachment extending into the interdental papilla), and papilla penetrating (frenal attachment crossing the alveolar process and extending up to the palatine papilla). Sewerin [12] identified the following types of frena: normal frenum, persistent tectolabial frenum, frenum with an appendix, frenum with nodule, duplication of the frenum, reces of the frenum, and bifid frenum.

Ten patients with a positive blanch test, inadequate vestibular depth, and midline diastema were diagnosed to have maxillary pathological frenal attachments [2, 12, 13]. These patients underwent frenectomy by scalpel method and 980 nm diode laser-assisted frenectomy. A single clinician, A1 (Author 1), performed all the surgical procedures.

### 2.1. Outcome Measures

The following parameters were assessed in all ten patients on days 1, 7, and 30 as outcome measures.

1. Patient-reported pain perception is recorded by Wong–Baker Faces Pain Rating Scale [14].
2. A wound healing assessment is made using the Early Wound Healing Index [15].

Five out of the ten patients opted for scalpel frenectomy. The procedure was explained, and informed consent was taken. Infiltration anesthesia is given in the vestibule region of teeth #11 and #21 with 2% lignocaine and 1 : 80,000 adrenaline. Frenectomy was performed by the classical method [16, 17]. Frenum was held using a hemostat at a maximum depth of the vestibule, and incisions were given with scalpel no. 15 at the upper and under the surface of the hemostat until the hemostat was free. Muscle fibers were detached from the bone. After blunt dissection, interrupted sutures were placed with Ethicon mersilk #3-0 braided suture. A periodontal pack was placed.

**Figure 1:**
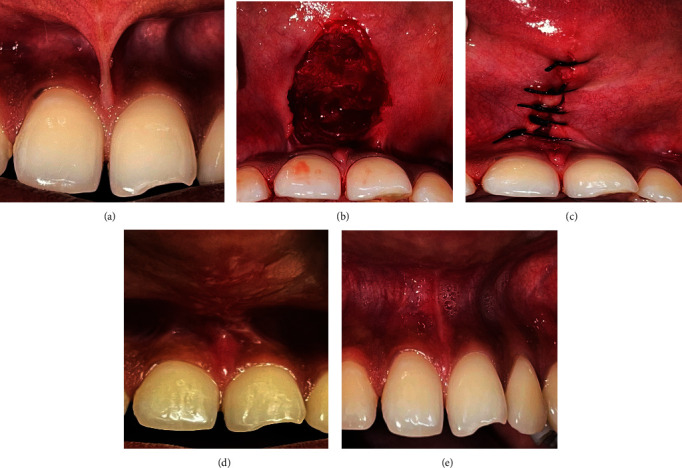
Clinical images of scalpel-treated patients on preoperative and postoperative days, 1, 7, and 30. (a) Preoperative. (b) Immediate postoperative. (c) Sutures placed. (d) 7-day postoperative. (e) 1-month postoperative.

**Figure 2:**
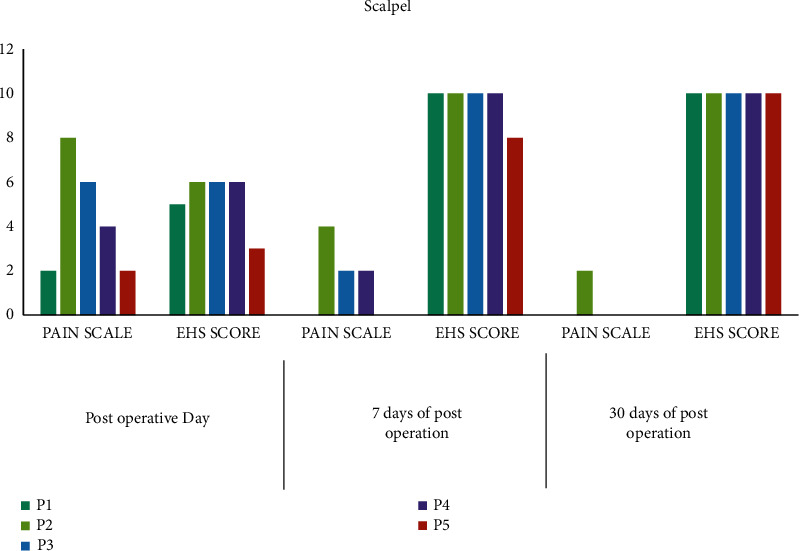
Pain scores of scalpel-treated patients.

The clinical photographs of scalpel frenectomy are illustrated in Figure 1. A summary of outcome measures for scalpel-treated patients is shown in Figure Error! Reference source not found Figure 2.

Another five patients opted for a laser-assisted frenectomy (laser device manufacturer: MDX-Medelux Co., Ltd., floor 3, no. 128, lane 928, zhennan road, Putuo District, Shangai, China. model number: DD-10, wavelength: 980 nm, output power: 0.5–10 W adjustable, laser class: Class 4). The patient, operator, and assistant used laser safety protective eyewear. The maxillary frenum region was anesthetized by infiltration anesthesia using 2% lignocaine and 1 : 80,000 adrenaline. Laser power settings were adjusted at 1 W continuous wave, contact mode, and a 300 *μ*m tip was moved in a paintbrush motion from the base to the apex of the frenum. Remnants of charred tissue were removed using sterile gauze dipped in saline, and the laser plume was removed with a high vacuum suction tip. Two of the five patients who underwent laser-assisted frenectomy did not require sutures and a periodontal pack. The other three patients required sutures as the area of the wound was large.

**Figure 3:**
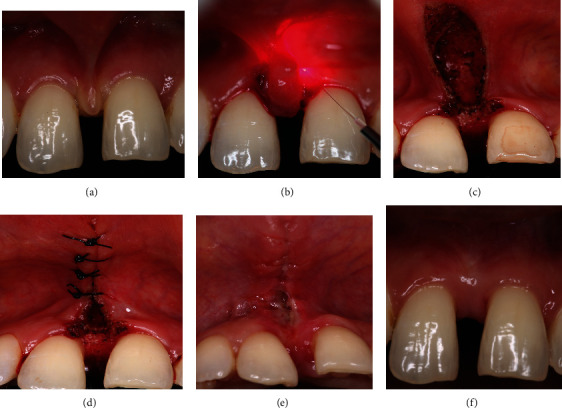
Clinical images of laser-treated patients on preoperative and postoperative days, 1, 7, and 30. (a) Preoperative. (b) 980 nm diode laser. (c) Immediate postoperative. (d) Sutures placed. (e) 7-day postoperative. (f) 1-month postoperative.

**Figure 4:**
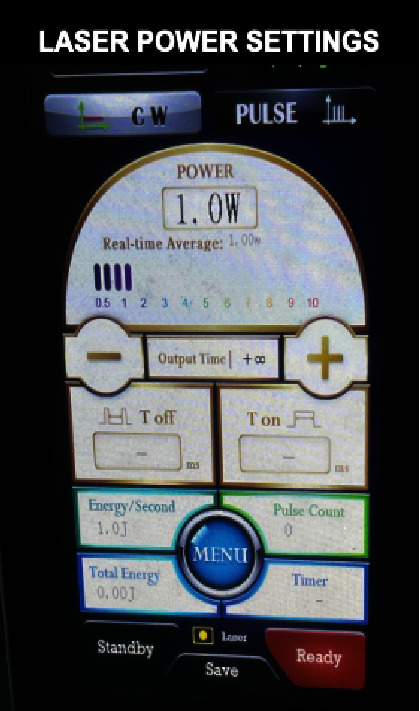
The laser unit with power settings.

**Figure 5:**
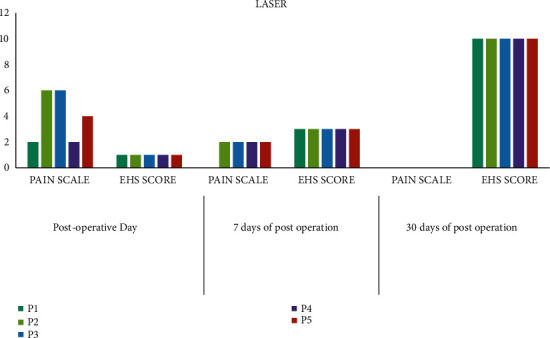
Pain scores of laser-treated patients.

Clinical images of the laser-assisted frenectomy procedure are illustrated in Figure 3. The laser unit with power settings is shown in Figure 4. A summary of outcome measures for laser patients is shown in Figure 5.

Among the scalpel-treated patients, on the postoperative days 1, 7, and 30, patient P1 gave pain scores of 2, 0, and 0, whereas the second patient, P2, gave pain scores of 8, 4, and 2. The third patient, designated as P3, gave pain scores of 6, 2, and 0. P4 gave pain scores of 4, 2, and 0, whereas P5 gave pain scores of 2, 0, and 0, respectively.

Among the laser-treated patients, on the postoperative days 1, 7, and 30, patient P1 gave pain scores of 2, 0, and 0; P2 gave pain scores of 6, 2, and 0; P3 gave pain scores of 6, 2, and 0; P4 gave pain scores of 2, 2, and 0; P5 gave pain scores of 4, 2, and 0, respectively.

In the scalpel-treated patients, early wound healing scores (EHS) were examined on postoperative days 1, 7, and 30. Here, P1 gave EHS scores of 5, 10, and 10; P2 gave EHS scores of 6, 10, and 10; P3 gave EHS scores of 6, 10, and 10; P4 gave EHS scores of 6, 10, and 10; P5 gave EHS scores of 3, 8, and 10, respectively.

Similarly, in the laser-treated patients, EHS scores were examined on postoperative days 1, 7, and 30. P1 gave EHS scores of 1, 3, and 10; P2 gave EHS scores of 1, 3, and 10; P3 gave EHS scores of 1, 3, and 10; P4 gave EHS scores of 1, 3, and 10; P5 gave EHS scores of 1, 3, and 10.

**Figure 6:**
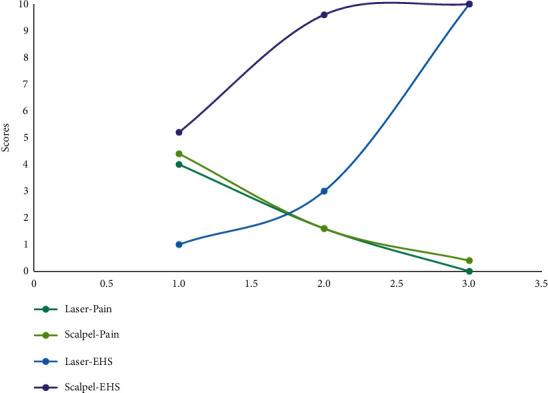
Average pain scores among all the patients.

Average pain scores among all the patients and a summary of outcome parameters are given in Figure 6 and Table 1, respectively.

From the above observations, pain scores in patients who underwent scalpel frenectomy were comparatively higher than in the laser-treated patients. In contrast, wound healing scores were higher in the scalpel group, demonstrating early wound healing in scalpel-treated patients and delayed wound healing in laser-treated patients.

## 3. Discussion

Management of high frenal attachments requires patient motivation and acceptance. Electrosurgery and lasers emerged as alternative treatment modalities to overcome the undesirable effects associated with scalpel frenectomy methods. Randomized clinical trials [5–10] comparing lasers to scalpel methods concluded that lasers provide a painless patient experience, apart from the numerous other advantages like a bloodless field, precision incision margins, and reduced chairside time [7, 11].

It was noted that the pain scores of scalpel-treated patients are higher than the laser-treated patients, reconfirming the results of previous studies [5–10]. A few limitations of the present case series are not being able to record the analgesic consumption in all the patients, not being able to record the time taken to complete the procedure, and ease of operation by the clinician, which could have added additional value to the available literature.

However, wound healing in laser-treated patients is observed to be delayed, consistent with a few studies [7, 18], and in contrast to a few studies [6, 8, 9]. The delayed wound healing following laser therapy is due to the thermal denaturation of the treated area, compromising the adhesion of the incised surfaces for primary closure [18].

Therefore, to overcome this drawback, a few studies [19] used adjuncts, like hyaluronic acid following laser therapy, to enhance wound healing. Hyaluronic acid is a promising agent to enhance wound healing in periodontal surgical procedures [20, 21].

The aspect of wound healing following laser therapy needs to be evaluated further in randomized controlled clinical trials using larger sample sizes and reliable wound healing assessment tools.

Evaluation of pain scores indicates that a diode laser is an effective alternative for frenectomy, offering patients a painless and bloodless experience. EHS indicated delayed wound healing in laser-treated patients, recommending a need for research in the direction of adjuncts for wound healing following laser therapy.

## Figures and Tables

**Table 1:** Summary of outcome parameters.

	Postoperative		7-day postoperative		30-day postoperative	
	Mean ± SD	Median (min–max)	Mean ± SD	Median (min–max)	Mean ± SD	Median (min–max)
Laser						
Pain score	4 ± 2	4 (2–6)	1.6 ± 0.89	2 (0–2)	0	0
EHS score	1 ± 0	1 (1–1)	3 ± 0	3 (3–3)	10	10 (10–10)
Scalpel						
Pain score	4.4 ± 2.6	4 (2–8)	1.6 ± 1.6	1.6 (0–4)	0.4 ± 0.89	0 (0–2)
EHS score	5.2 ± 1.3	5.2 (3–6)	9.6 ± 0.89	10 (8–10)	10	10 (10–10)

## Data Availability

All the data, including tables and figures, are included in the manuscript.
